# Variation in North American Infectious Disease Specialists' Practice Regarding Oral and Suppressive Antibiotics for Adult Osteoarticular Infections: Results of an Emerging Infections Network (EIN) Survey

**DOI:** 10.1093/ofid/ofae280

**Published:** 2024-05-15

**Authors:** Nicolás Cortés-Penfield, Susan E Beekmann, Philip M Polgreen, Keenan Ryan, Jonas Marschall, Poorani Sekar

**Affiliations:** Division of Infectious Diseases, University of Nebraska Medical Center, Omaha, Nebraska, USA; Division of Infectious Diseases, Carver College of Medicine University of Iowa, Iowa City, Iowa, USA; Division of Infectious Diseases, Carver College of Medicine University of Iowa, Iowa City, Iowa, USA; Inpatient Pharmacy Department, University of New Mexico Hospital, Albuquerque, New Mexico, USA; Division of Infectious Diseases, Washington University School of Medicine, St. Louis, Missouri, USA; Division of Infectious Diseases, Carver College of Medicine University of Iowa, Iowa City, Iowa, USA

**Keywords:** chronic suppresive antibiotic therapy, infectious disease practice, oral antibiotic therapy, osteoarticular infections, practice guidelines

## Abstract

**Background:**

Osteoarticular infections (OAIs) are commonly treated with prolonged intravenous (IV) antimicrobials. The Oral versus Intravenous Antibiotics for Bone and Joint Infection (OVIVA) trial demonstrated that oral (PO) antibiotics are noninferior to IV antibiotics in the treatment of OAIs. We surveyed infectious disease (ID) physicians about their use of PO antibiotics in the treatment of OAIs.

**Methods:**

An Emerging Infection Network survey with 9 questions regarding antibiotic prescribing for the treatment of OAIs was sent to 1475 North American ID physicians. The questions were mostly multiple choice and focused on the use of definitive oral antibiotic therapy (defined as oral switch within 2 weeks of starting antibiotics) and chronic suppressive antibiotic therapy (SAT).

**Results:**

Of the 413 physicians who reported treating OAIs, 91% used oral antibiotics at least sometimes and 31% used them as definitive therapy, most often for diabetic foot osteomyelitis and native joint septic arthritis. The oral antibiotics most frequently used for OAIs included trimethoprim-sulfamethoxazole, doxycycline/minocycline, and linezolid for *Staphylococcus aureus*, amoxicillin/cefadroxil/cephalexin for streptococci, and fluoroquinolones for gram-negative organisms. The most common rationales for not transitioning to oral antibiotics included nonsusceptible pathogens, comorbidities preventing therapeutic drug levels, and concerns about adherence. SAT use was variable but employed by a majority in most cases of periprosthetic joint infection managed with debridement and implant retention.

**Conclusions:**

North American ID physicians utilize oral antibiotics and SAT for the management of OAIs, although significant practice variation exists. Respondents voiced a need for updated guidelines.

Nearly 1 million arthroplasties are performed in the United States each year, and this number is projected to increase to 2–3.5 million by 2030 [1–3]. Prosthetic joint infection (PJI) complicates 0.5%–2% of arthroplasties and is associated with high morbidity, mortality, and cost [4, 5]. Treatment failure rates for PJIs are high and highly variable; a recent systematic review including 99 studies found the average treatment failure rate in PJI managed with debridement, antibiotics, and implant retention (DAIR) to be 38.6%, ranging from 0% to 88.9% [6]. Five-year mortality rates following PJI exceed 20% [7]. Other osteoarticular infections (OAIs) have similarly poor prognoses; for example, among patients with diabetes-related foot infection (DFI) severe enough to require hospitalization, up to half eventually require amputation [8], after which the 5-year mortality ranges from 46% to 56% [9]. Treatment of fracture-related infection (FRI) cases generates 6.5 times higher health care costs than noninfected fracture cases, with a reported success rate of only 70% [10]. Updated approaches to OAI prevention and management are needed.

The Infectious Disease Society of America (IDSA) published clinical practice guidelines for the management of PJI in 2013 and currently considers them archived [11]; for other OAIs, such as native joint septic arthritis and FRI, no IDSA guidelines exist. Over the past decade, results from randomized controlled trials (RCTs) regarding the use of oral antibiotics in OAIs, the optimal duration of antimicrobial therapy for a range of OAIs [12–15], the value of secondary antimicrobial prophylaxis following 2-stage exchange for PJI [16], and the value of rifampin in staphylococcal PJI [17] have become available. Because these data have yet to be incorporated into updated IDSA guidelines, to what extent they have already been adopted into clinical practice in the United States remains unclear.

Before the publication of IDSA guidelines for OAIs, infectious disease (ID) specialists' treatment approaches for these infections varied substantially; however, this variation has not been reassessed in over a decade [18]. We surveyed ID specialists about their approach to PJI and other complex osteoarticular infections, focusing on 2 key practice domains: (1) use of oral antimicrobials for different types of bone and joint infections and (2) use of chronic suppressive antibiotic therapy (SAT; generally defined as antibiotic use extended beyond the 3–6 months of primary treatment recommended in IDSA guidelines following DAIR or single-stage exchange) or secondary antibiotic prophylaxis in patients with prior PJI (defined as antibiotics given after a 2-stage exchange surgery anywhere from 2 weeks to 3 months after the prothesis is re-implanted to prevent another infection).

## METHODS

We distributed a survey about osteoarticular infections (OAIs) via the IDSA's Emerging Infection Network (EIN), a group of ∼1500 adult ID physicians from North America who have volunteered to participate in regular surveys regarding ID clinical practice. We defined OAIs as diabetes-related foot osteomyelitis, other osteomyelitis (eg, of the extremity or spine) with or without associated hardware, native joint septic arthritis, and prosthetic joint infection.

Two ID physicians and 1 ID pharmacist who share a clinical focus in orthopedic infections (N.C., P.S., and K.R.) developed the initial survey questions, which were then refined in collaboration with the EIN leadership (S.B. and P.P.). Four questions focused on when, in whom, and with what rationale clinicians selected oral vs intravenous antibiotics for OAI. Three questions focused on patient selection for either SAT or secondary prophylaxis following surgery for PJI. The survey instrument is included in the Supplementary Data.

We sent 3 requests to complete the survey between November and December 2022. Denominators varied because not all EIN members responded to all questions. For some questions, respondents could select >1 option, resulting in some percentages totaling >100%. We performed descriptive statistics and assessed statistical significance using χ^2^ tests, considering *P* = .05 the threshold of significance.

## RESULTS

Of the 1475 EIN members who had ever responded to an EIN survey, 490 (33%) submitted responses, of whom 77 reported not seeing patients with OAIs and opted out of the survey. The remaining 413 respondents practiced in all US Census Bureau divisions, with the Pacific (18%), South Atlantic (15%), Mid-Atlantic (13%), and East North Central (13%) divisions best represented. Respondents most commonly practiced in university (35%), community (28%), or nonuniversity teaching hospitals (25%); 7% practiced in the Veteran's Health Administration or Department of Defense systems. Most respondents had numerous years of clinical experience: 26% were >25 years out from ID fellowship, 17% were 15–24 years out, 40% were 5–14 years out, and 17% were <5 years out from ID fellowship. These respondents most often reported seeing 6–10 admitted patients with osteoarticular infections a month (43%), followed by 11–20 such patients (23%), <5 such patients (20%), or >20 such patients (14%). The most reported monthly outpatient volume was <5 patients with osteoarticular infections (49%), followed by 6–10 patients (29%), 11–20 patients (15%), and >20 patients (7%).

**Figure 1.**
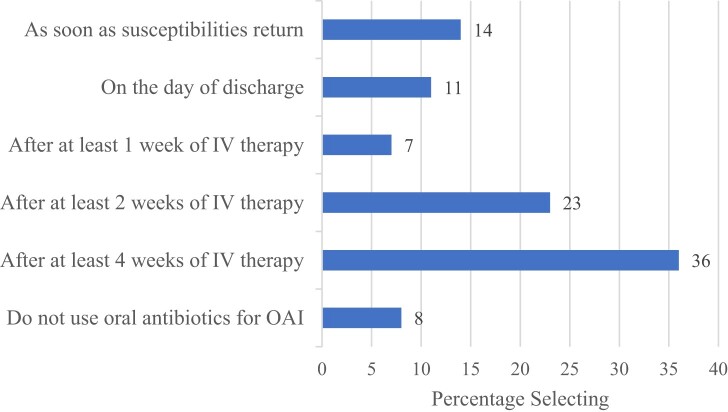
When do you switch from IV to oral antibiotics for osteoarticular infections? Abbreviations: IV, intravenous; OAI, osteoarticular infection.

**Figure 2.**
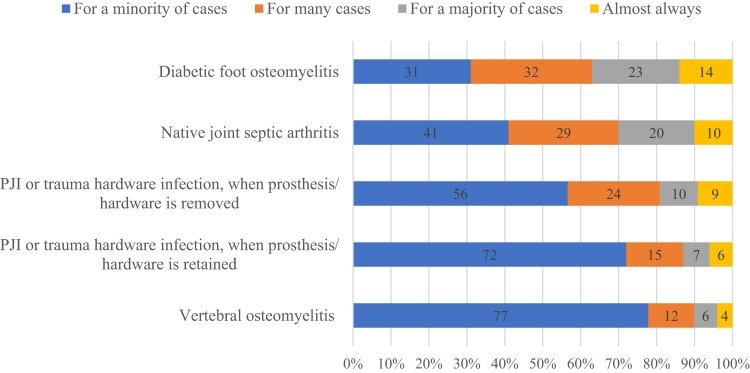
For the following infections, how often do you use oral antibiotics as definitive therapy (ie, switch to PO within 2 weeks of starting antibiotics)? Abbreviations: PJI, prosthetic joint infection; PO, per os (oral).

Most respondents (376/413, 91%) reported using oral antibiotics for at least some osteoarticular infections, but only 129/413 (31%) used oral antibiotics as definitive therapy, which we defined as switching from IV to oral antibiotics within 2 weeks of starting antimicrobials (Figure 1). Respondents were most likely to routinely employ oral antibiotics as definitive therapy for diabetes-related foot osteomyelitis (69%) and native joint septic arthritis (59%); fewer respondents reported using oral agents for definitive therapy in orthopedic hardware infections managed with device removal (43%) or retention (28%), or for vertebral osteomyelitis (22%) (Figure 2; these percentages were calculated with the respondents indicating they do not use oral agents for osteoarticular infections in the sample).

A majority of respondents supported routine use of trimethoprim-sulfamethoxazole (71%), doxycycline or minocycline (68%), or linezolid (63%) as oral definitive therapy for OAI due to *Staphylococcus aureus*; amoxicillin, cephalexin, or cefadroxil (72%) for streptococci; and fluoroquinolones (84%) for gram-negative bacilli. No specific antibiotic agent was preferred by a majority for *Cutibacterium acnes* OAI, but respondents favored the use of amoxicillin, cephalexin, or cefadroxil (43%) and doxycycline or minocycline (33%). In the open response section, small minorities (1%–3%) advocated routine use of clindamycin for gram-positive pathogens or later-generation oral cephalosporins for gram-negative pathogens.

**Figure 3.**
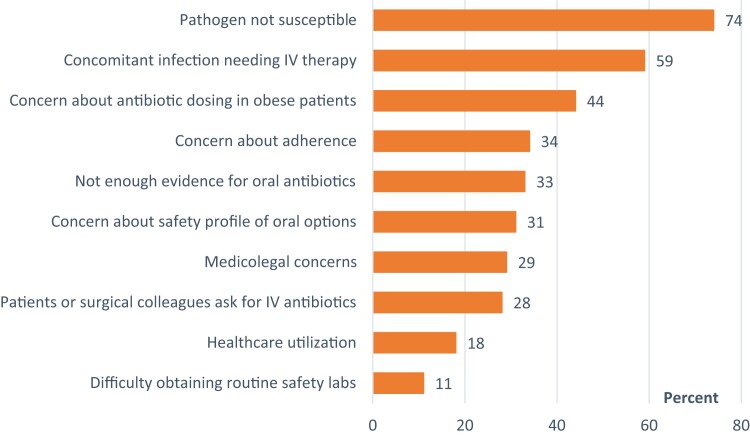
When you choose NOT to use oral antibiotics in osteoarticular infections, which of these factors *most strongly* drives your decision? (Additional instructions to respondents: Select the top 3 reasons for this decision.) Abbreviation: IV, intravenous.

Respondents provided multiple reasons for not using oral antibiotics (note: while the survey asked respondents to select no more than 3 reasons from the list provided, 39% of respondents selected 4 or more options), with the majority selecting both lack of an active oral agent and coinfection perceived to require IV therapy (eg, concurrent infective endocarditis) (Figure 3). Other reasons commonly selected included concerns about use of oral antibiotics in obese patients, patient adherence to oral antibiotic regimens, oral antibiotics' safety profile, and medicolegal issues. In an open text field, respondents also expressed concerns about specific pathogens (n = 5), bioavailability of certain oral antibiotics (n = 5), adequacy of oral antibiotics in patients with suboptimal source control (n = 4), tolerance of high-dose oral antibiotics (n = 3), the institutional practice of peers (n = 3), and the perceived lack of clinical guidelines supporting the use of oral antibiotics (n = 2).

**Figure 4.**
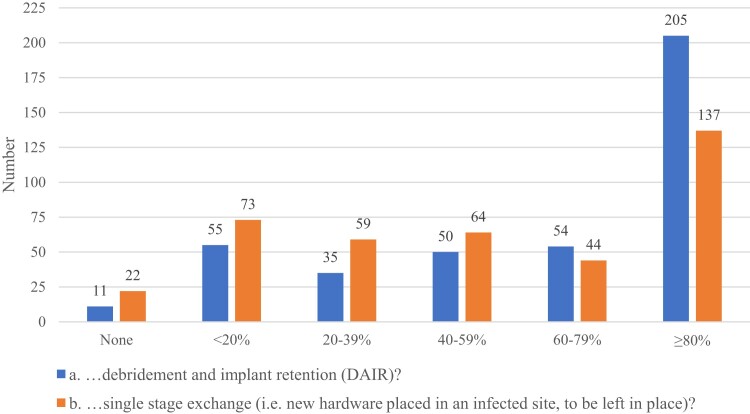
What percentage of these patients do you place on SAT after DAIR and single-stage exchange? (SAT was defined as antibiotics given beyond the 3–6 months recommended by IDSA guidelines for initial treatment of PJI.) Abbreviations: DAIR, debridement, antibiotics, and implant retention; IDSA, Infectious Diseases Society of America; PJI, prosthetic joint infection; SAT, suppressive antibiotic therapy.

A majority of respondents reported using SAT in most patients with PJI managed with DAIR (Figure 4). Responses were much more heterogeneous regarding use of SAT following single-stage exchange for PJI, with use of SAT in ≥80% of patients being less common (reported by 137/413 [33%] respondents for single-stage exchange vs 205/413 [50%] for DAIR). When initiating SAT, many respondents intended it as lifelong therapy (40%), with 1 year being the second most common duration (24%) and a small minority basing the decision on follow-up studies such as inflammatory markers (5%) or repeat radiographs (7%). Ninety-three respondents provided open text field “other” answers reflecting a wide range of practice; the most common of these included some version of “it depends” (n = 12), a 3–6-month duration of therapy (n = 10), and variable durations of suppression based on the pathogen (n = 10), patient factors (n = 7), or other issues (n = 6).

There was no consensus about the use of secondary antimicrobial prophylaxis following 2-stage exchange for PJI, which is based on a recent multicenter randomized controlled trial suggesting that extended courses of oral antibiotics might prevent new episodes of PJI in this high-risk patient population [15]. Sixty percent of respondents stated that they do not routinely use such prophylaxis; the next most common strategies were antimicrobial prophylaxis directed toward the previously isolated pathogen for either 2–4 weeks (16%) or 3 months (14%). The 25 respondents who described their approach in an open text field provided a wide range of answers, including prophylaxis until operative cultures finalize as negative (n = 4), and some variation of “the orthopedic surgeons give antibiotics without seeking our input” (n = 3).

Finally, 82 respondents answered the concluding open text field question with further comments on osteoarticular infections. The most common opinion, expressed by 14 EIN member physicians, was that OAI treatment decisions need to be highly individualized based on patient, pathogen, and surgical factors; 9 members felt the survey questions were insufficient in scope, specificity, and/or range of responses to adequately capture this nuance. The second key theme that emerged was a desire for updated society guidelines, expressed by 8 members; respondents expressed the desire to use more oral antibiotics but reported pushback from surgeons or patients who requested IV antibiotics (n = 7), medicolegal concerns about oral antibiotics not being the standard of care (n = 4), and fear of practicing differently from local peers (n = 3). Other common themes included uncertainty about the value or optimal duration of SAT (n = 5), bemoaning the lack of quality data in the management of orthopedic infections (n = 5), emphasizing the importance of source control (n = 4), and expressing appreciation for the survey and/or curiosity about the results (n = 4).

## DISCUSSION

We found that North American adult ID physicians' approaches for treating PJIs and other OAIs were highly variable and differed by infection site and type. Most respondents reported using oral antibiotics for at least some bone and joint infections, but only a minority used oral agents for a major portion of the treatment course, with a third of the respondents (36%) routinely continuing IV antibiotics for at least 4 weeks. Respondents were more likely to use oral antibiotics in diabetes-related foot infections and native joint infections compared with vertebral osteomyelitis or infections involving orthopedic hardware, particularly when the hardware is retained.

Respondents also reported a range of different treatment approaches regarding the use of SAT. A majority of respondents employed SAT for patients with PJI managed with hardware retention, with 40% of respondents usually prescribing SAT as lifelong therapy and approximately a quarter usually limiting the duration of SAT to 1 year. This figure appears to be similar to prior reports using the same network of infectious disease physicians. For example, 36% recommended lifelong therapy in a 2010 study [19], and 41% in 2013 [18]. Data on the efficacy of SAT in PJI after DAIR are limited to small, heterogenous observational studies, some of which suggest the first 12 months may be the primary period of benefit [20–22]. Due to survey size constraints, we did not inquire about respondents' use of SAT in other orthopedic device infections managed with hardware retention (eg, FRIs and vertebral hardware infections). Data are far more limited for SAT in these infections vs PJI, but anecdotally SAT is also commonly employed in these clinical scenarios, and presumably the same biological principles would apply.

A recent randomized controlled trial supports the use of antibiotics after 2-stage exchange [16]. However, we found that 60% of respondents do not routinely prescribe antibiotics after reimplantation. This result may indicate that studies originally published in the orthopedic literature may not yet be widely disseminated across ID physician members of the EIN or that confidence in this trial's findings is low in the context of prior studies finding no benefit to postoperative antibiotic prophylaxis in other settings.

Multiple randomized controlled trials and observational studies have reported equivalent efficacy of oral and IV antibiotic regimens for bone and joint infections, with the largest and most widely referenced OVIVA trial concluding that oral antibiotics were noninferior to IV antibiotics when used in the first 6 weeks for the treatment of bone and joint infections, as assessed by treatment failure at 1 year [23]. Approximately 10 years ago, we asked physician members of the EIN about switching from IV to oral antibiotics, and we found that this was not a common practice: 84% of respondents reported that they did not switch patients from IV to oral antibiotics during a treatment course [18]. In contrast, only 8% of respondents in our current study reported that they did not at times switch from IV to oral antibiotics for these infections, suggesting an evolving practice pattern in North America. However, despite the increased use of oral regimens, we found that the use of prolonged IV antibiotics for bone and joint infection is still prevalent, with the most common duration of IV therapy being 4 weeks (Figure 1).

The free-text comments by respondents highlighted several rationales for at least a situational preference for IV over oral antibiotics. First, some respondents preferred IV therapy in cases where higher drug concentrations might be beneficial, for example, patients with poor vascular supply or incomplete source control. Other respondents perceived that bioavailability and susceptibility issues of specific organisms limited the role for at least some oral antibiotics for some pathogens. Additional concerns revolved around potential problems tolerating long courses of oral therapy as well as concerns about adherence to oral regimens compared with IV therapy. While self-reported adherence to oral therapy in OVIVA was indeed slightly lower in patients randomized to PO vs IV (87.6% vs 93.8% reporting either medium or high adherence), >90% of patients given PO therapy via a Medication Event Monitoring System adhered to at least 95% of doses, and most importantly, clinical outcomes with IV and PO remained similar [23]. Early treatment discontinuation in OVIVA was more common in the IV subgroup (18.9% vs 12.8%; *P* = .006), likely driven by IV catheter complications. Conversely, a pre–post implementation of OVIVA trial findings into clinical practice in an orthopedic hospital in the United Kingdom found an increased rate of adverse drug reactions in the postimplementation group with increased utilization of oral antibiotics (23% vs 36.2%), which ultimately led to a discontinuation rate 1.5 times greater than in the pre-implementation group, driven by gastrointestinal intolerances (9% vs 24%) [24].

Logistical and follow-up issues may also play a role in treatment decisions. For example, while patients on IV therapy are often followed closely via institutional outpatient parenteral antimicrobial therapy (OPAT) teams, similar follow-up mechanisms are often not in place for patients discharged on oral antibiotics. Furthermore, due to staffing issues, some members reported difficulty seeing patients in a timely manner following their hospital discharge and considered this a barrier to using or switching to oral agents.

Multiple respondents highlighted the need for additional larger studies, noting that it is sometimes difficult to generalize the results of trials to patients who might have been excluded. Finally, some respondents noted that treatment decisions for orthopedic infections were often shared with surgeons and that the surgeons were frequently reluctant to use oral antibiotics. Publication of treatment guidelines endorsed jointly by the ID and orthopedic societies may help with those shared decisions; indeed, the need for new and up-to-date treatment guidelines was a common theme in the comment sections.

While adoption of secondary antibiotic prophylaxis after exchange arthroplasty remains limited, we found that clinicians often use long-term, frequently lifelong, SAT after DAIR or single-stage exchange. This practice, while mentioned as an option in the 2013 IDSA PJI guidelines, is based on limited, methodologically heterogenous, and confounded observational data [22]. Prolonged antibiotic exposure can lead to adverse events and the emergence of resistant pathogens. Defining the appropriate use of SAT in PJI should be considered an urgent antimicrobial stewardship issue and subject to prospective trials.

Our study has several limitations. First, as per the EIN's approach and to achieve high response rates, the survey was relatively short. As a result, we were unable to fully explore the reasons for practice variation. This frustrated several of our respondents, and a longer survey with different clinical scenarios could have better captured the nuances of respondents' practices. Second, these data reflect EIN members' self-reported practice behaviors rather than direct review of their clinical care. Finally, this survey was focused on primarily US-based ID physicians and does not reflect the practice patterns elsewhere (eg, Europe, where early transition to oral antibiotics for osteoarticular infections has been a longstanding practice).

In conclusion, ID physicians’ practices regarding the treatment of orthopedic infections are highly variable between providers and are also highly individualized to specific patients and types of infections. Our results, compared with prior surveys of the same network, appear to indicate that the use of oral antibiotics for OAI is becoming more common. However, the results of some recent randomized clinical trials appear not to be fully adopted in many clinical practices. We advocate for new interdisciplinary guidelines endorsed by the IDSA to help inform clinical practice using existing literature and highlight the need for additional research.

## Supplementary Material

ofae280_Supplementary_Data

## Supplementary Data

Supplementary materials are available at *Open Forum Infectious Diseases* online. Consisting of data provided by the authors to benefit the reader, the posted materials are not copyedited and are the sole responsibility of the authors, so questions or comments should be addressed to the corresponding author.
