# Supplementary material for: Variation in North American Infectious Disease Specialists' Practice Regarding Oral and Suppressive Antibiotics for Adult Osteoarticular Infections: Results of an Emerging Infections Network (EIN) Survey
Source: Open Forum Infect Dis. 2024 May 15;11(6):ofae280. doi: 10.1093/ofid/ofae280 (PMC11167670; doi:10.1093/ofid/ofae280)
Supplement: ofae280_Supplementary_Data [file ofae280_supplementary_data.pdf]

INFECTIOUS DISEASES SOCIETY OF AMERICA EMERGING INFECTIONS NETWORK QUERY:

Management of Osteoarticular Infections

Traditionally, osteoarticular infections in the U.S. have been treated with intravenous (IV) antibiotics for 4 to 6 weeks. The Oral versus Intravenous Antibiotics for Bone and Joint Infection (OVIVA) trial was published in 2019 (DOI: 10.1056/NEJMoa1710926) and showed oral antibiotics were non inferior to IV antibiotics in the treatment of these infections. The purpose of this survey is to gauge practice patterns of ID specialists in the treatment of osteoarticular infections and potential concerns/ barriers they may have in using oral antibiotics. We would also like to learn about the pattern of use of suppressive antibiotic therapy in hardware-associated infections given the paucity of published studies on this topic.

The purpose of this survey is to assess current practice patterns of ID providers who care for patients with a variety of osteoarticular infections.

Osteoarticular infections include:
- osteomyelitis
- native joint septic arthritis
- prosthetic joint infections (PJI)
- trauma-related orthopedic hardware infections

Please fax to 319-384-8860 OR 1-866-890-5964

EMERGING INFECTIONS NETWORK QUERY
Management of Osteoarticular Infections

Name: ________________________________

1. In an average month, how many unique patients with osteoarticular infections do you see…

a. …in the hospital? __ ≤ 5 __ 6 to 10 __ 11 to 20 __ More than 20

b. …in the clinic? __ ≤ 5 __ 6 to 10 __ 11 to 20 __ More than 20

__ Do not routinely care for these patients in either setting -- *please* STOP HERE

Use of Oral Antibiotics (other than rifampin)

2a. When you use oral antibiotics in osteoarticular infections, when do you switch from IV to PO? (assume surgical debridement was performed, with retention of any infected hardware)

__ As soon as susceptibilities return and the patient can tolerate PO (i.e. no minimum duration of IV)
__ On the day of hospital discharge (i.e. no minimum duration of IV)
__ After at least 1 week of IV therapy
__ After at least 2 weeks of IV therapy
__ After at least 4 weeks of IV therapy
__ I do not use oral antibiotics for osteoarticular infections

2b. If infected hardware was removed, would this change your answer? __ Yes __ No

3. For the following infections, how often do you use oral antibiotics as definitive therapy (i.e. switch to PO within 2 weeks of starting antibiotics)?

	For minority of cases (0-24%)	For many cases (25-49%)	For a majority of cases (50-74%)	Almost always (75-100%)
Native joint septic arthritis	__	__	__	__
Vertebral osteomyelitis	__	__	__	__
Diabetic foot osteomyelitis	__	__	__	__
PJI or trauma hardware infection, when the prosthesis/ hardware is removed	__	__	__	__
PJI or trauma hardware infection, when the prosthesis/ hardware is retained	__	__	__	__

4. Which oral antibiotics do you routinely use for definitive therapy of osteoarticular infections (i.e. switching to orals within 2 weeks of starting antibiotics) due to the following pathogens?

[Select any that apply]	*S. aureus*	Coag-negative staphylococci	Strepto-cocci	GNRs	*C. acnes*
Fluoroquinolones (combined w rifampin for staphylococci)	__	__	__	__	__
Trimethoprim-sulfa	__	__	__	__	__
Doxycycline or minocycline	__	__	__	__	__
Linezolid	__	__	__	__	__
Amoxicillin, cephalexin or cefadroxil	__	__	__	__	__
Other oral agent: ___________	__	__	__	__	__

Please fax to 319-384-8860 OR 1-866-890-5964

5. When you choose NOT to use oral antibiotics in osteoarticular infections, which of these factors most strongly drive your decision? *[Select the top three reasons for this decision]*

___ Not enough evidence for oral antibiotics
___ Concomitant infection needing IV therapy (e.g. infective endocarditis)
___ Identified pathogens not susceptible
___ Comorbidities preventing therapeutic drug levels with oral antibiotics (e.g. BMI>40)
___ Concern about oral options' safety profile (e.g. collagen-vascular side effects)
___ Concern about adherence to oral antibiotics
___ Difficulty obtaining routine safety labs after discharge on oral antibiotics
___ Patients or surgical colleagues ask for IV antibiotics
___ Healthcare utilization (i.e. continuing IV antibiotics allows access to additional services)
___ Medicolegal concerns (i.e. accusation of substandard care with bad outcome)
___ Other, *specify*:

Suppressive Antibiotic Therapy for PJI or Other Orthopedic Hardware Infection (i.e. antibiotics given beyond the 3-6 months indicated for initial treatment of PJI)

6. What percentage of these patients do you place on suppressive antibiotic therapy after…

a. … debridement and implant retention (DAIR)?

__ None __ <20% __ 20-39% __ 40-59% __ 60-79% __ 80-100%

b. … single stage exchange (i.e. new hardware placed in an infected site, to be left in place)?

__ None __ <20% __ 20-39% __ 40-59% __ 60-79% __ 80-100%

7. If you use suppressive antibiotic therapy for this purpose, what do you usually recommend as the duration of suppression?

__ N/A, do not use suppressive antibiotics
__ 1 year
__ Until inflammatory markers have normalized
__ Until complete bone healing is evident on plain radiographs
__ Lifelong, unless the implant is removed
__ Other, *describe:*

8. How do you manage antibiotic prophylaxis following reimplantation in PJI managed with 2-stage exchange (presuming intraoperative cultures at reimplantation are negative)?

__ I do not routinely prescribe antibiotics after reimplantation
__ I give antibiotics directed toward the originally cultured pathogen for 3 months
__ I give a standard/protocolized antibiotic (i.e. not directed to prior cultures) for 3 months
__ I give antibiotics directed toward the original cultured pathogen for 2-4 weeks
__ I give a standard/protocolized antibiotic (i.e. not directed to prior cultures) for 2-4 weeks
__ Something else, *describe:*

9. Any comments about management of osteoarticular infections?

Thank you for completing this survey!

Please fax to 319-384-8860 OR 1-866-890-5964
